# Development of a patient assessment to meet the needs of patients suffering from advanced non-oncological diseases – the KOPAL trial

**DOI:** 10.1186/s12875-025-02750-z

**Published:** 2025-02-22

**Authors:** Gabriella Marx, Tina Mallon, Henrikje Stanze, Manuel Zimansky, Nils Schneider, Friedemann Nauck, Martin Scherer, Nadine Pohontsch

**Affiliations:** 1 Department of General Practice and Primary Care, University Medical Center Hamburg-Eppendorf, Martinistr. 52, Hamburg, 20246 Germany; 2 Department 3, Hochschule Bremen, City University of Applied Sciences, Bremen, Germany; 3 Department of Palliative Medicine, University Medical Center Goettingen, Goettingen, Germany; 4 Institute for General Practice and Palliative Care, Hannover Medical School, Hannover, Germany

**Keywords:** Provider-patient communication, Decision-making, Primary care, Palliative care

## Abstract

**Background:**

Patients suffering from progressive non-oncologic chronic diseases are primarily treated in primary care. Early integration of palliative care (PC) can improve patients’ quality of life and reduce burdensome physical symptoms. To guide interprofessional counselling between GPs and specialist palliative home care teams, we developed an aide memoire for patients diagnosed with advanced non-oncological chronic diseases, the KOPAL conversation guide, as part of the KOPAL trial. The aim of this study was to ensure the conversation guide covers all relevant care aspects in order to reveal individual gaps and needs in healthcare.

**Methods:**

We conducted three focus groups including four patients, seven health care providers, and five stakeholders. During each group, a draft of the conversation guide was discussed, revised, and consented from the respective perspectives.

**Results:**

The final KOPAL conversation guide contains eight key topics: living with the illness, physical, emotional, personal, and social situation, information and communication, control and autonomy, emergency management. Each topic refers to a number of related subtopics listed in each respective thematic section. The conversation should start with the introductory question referring to the patient’s current well-being. At the end, patients are asked to state their primary concern based on the conversation.

**Conclusions:**

The KOPAL conversation guide is a broad evaluation and communication tool. It covers potential PC needs of non-oncological patients and provides a basis for interprofessional case planning, and counselling. Applying the guide may help to bridge gaps in communication between general and specialist PC professionals as well as between professionals and patients.

**Supplementary Information:**

The online version contains supplementary material available at 10.1186/s12875-025-02750-z.

## Background

Demographic change is leading to a growing number of elderly with serious chronic conditions majorly challenging health care in general, and end-of-life care in particular [1, 2]. Non-oncological chronic conditions are characterized by their long duration, slow progression, and prognostic uncertainty [3]. Patients suffering from progressive non-oncologic chronic diseases are mainly treated in primary care [4]. In Germany, outpatient palliative care (PC) is structurally separated in two areas: general PC provided by general practitioners (GP), and specialist palliative care (SPC) provided by trained doctors, nurses, and other professionals.

Research shows evidence that the early integration of PC can improve patients’ quality of life, reduce physically burdensome symptoms, increase advance directives, and improve caregiver burden, as well as patient and family satisfaction [5]. However, Davis et al. [5] point to the missing definition of ‘early’ palliative care, but “it does appear that for full benefits of palliative care to be realized, continuity by a multidisciplinary team is needed for at least 3–4 months.” A retrospective cohort study including 4650 patients conducted from 2012 to 2014 in the UK showed a median PC duration of 34 days (MAD = 29 days), whereas PC duration deviated up to 29 day in half of the study population. Median duration of PC was also longer in cancer patients than in patients with other conditions (MAD = 31 days vs. MAD = 14 days) [6]. Similarly, a systematic review from 2020 including 169 studies reported a median duration from PC initiation to death of less than 19 days, with longer PC duration for cancer patients compared to non-oncological patients [7]. Therefore, improvement of PC initiation is still needed, especially for non-oncological patients [8]. In primary care, chronic conditions related to the cardiovascular system (e.g., congestive heart failure (CHF), coronary heart disease, hypertension), the nervous system (mainly dementia), and the respiratory system (mainly chronic obstructive lung disease) are among the most common chronic diseases in Germany and worldwide [3, 9].

While the course of patients suffering from progressive non-oncological chronic diseases is often characterized by minor limitations in everyday life over a long period of time followed by serious episodes of illness or crises [1, 10], symptoms of distress are similar in patients diagnosed with chronic cardiorespiratory diseases to cancer patients at an advanced stage of the disease [11]. These illnesses typically follow two trajectories of functional decline described by Lynn and Adamson [1], empirically validated by Lunney et al. [12]: 1) Long-term limitations with intermittent exacerbations and sudden dying typically experienced in organ and system failure, and 2) prolonged dwindling as seen in dementia, disabling stroke, and frailty impeding GP’s to identify the appropriate time of PC initiation.

Screening tools to identify patients with potential PC needs generally “use prediction of death and/or deterioration as a proxy for the identification” of patients with unmet PC needs [13]. Against this background, applying a single screening tool only may not be sufficient. Rather, a timely interprofessional and interdisciplinary counselling between GPs and multidisciplinary specialist palliative home care teams (SPHC) using an aide memoire for non-oncological patients, e.g., the 'KOPAL conversation guide' could complement the counselling. The KOPAL trial is a multi-centre, two-arm, cluster randomised controlled trial (RCT) and is described elsewhere [14]. In brief, we conducted the study in three steps: 1) Development of the KOPAL conversation guide, 2) intervention (home visit by SPHC nurse and conversation with the patient followed by a brief SPHC team consultation and an interprofessional telephone case conference between GP, SPHC nurse, and SPHC doctor to discuss the patient’s health and care situation and needs) and quantitative investigation at baseline and four follow-up points within 48 weeks, 3a) health economic analyses and 3b) qualitative evaluation of the KOPAL-intervention. In this paper, we describe the development of the KOPAL conversation guide (step 1). The KOPAL conversation guide was used by the SPHC nurse during the home visit and, if desired, for the interprofessional telephone case conference (step 2). SPHC nurses received a full online training on the use of the guide beforehand. In step 3b, the intervention, including the application of the guide, was evaluated.

## Methods

### Study aim and design

The KOPAL conversation guide aimed to cover relevant care aspects and to reveal individual gaps and needs in healthcare. It has been developed as part of the KOPAL-trial [14] and will be presented in this paper. The process of development was guided by the following question: Which key topics should be addressed to fully identify possible palliative care needs of patients suffering from progressed non-oncological chronic diseases?

### Development of the KOPAL conversation guide

#### Preparation

The KOPAL conversation guide is based on the British ‘PEPSI COLA aide memoire’ (used with permission from the National GSF Centre in End of Life Care) a holistic patient assessment covering 9 core topics: Physical, Emotional, Personal, Social Support, Information/Communication, Control and Autonomy, Out of Hours, Living with illness, and After Care. The aide memoire includes numerous topics for consideration, related cue questions, and resources [15].

At first, a German version of the PEPSI COLA aide memoire was drafted eliminating all cancer related issues since the KOPAL conversation guide focuses on non-cancer patients. After producing a translated version, three of the study sites (Hamburg, Hannover, and Goettingen) repeatedly discussed the draft of the KOPAL conversation guide with palliative care experts (researchers and carers) in an iterative process and consented discussed items with special focus on practice, structure of palliative care, and legal requirements within the German healthcare system. Additionally, all items were compared to the German Guideline Palliative Care (S3-Leitlinie Palliativmedizin). The following dimensions therein – subjective-individual needs, objectively comprehensible demands, and material or individual resources – were integrated in the KOPAL conversation guide. In accordance with the practice of professional organisations in Germany (e.g., German Association for Hospice and Palliative Care, German Association for Palliative Medicine, Advance Care Planning Germany) a genogram was added as an optional tool to explore the social situation of the patients. After this process the draft of the KOPAL conversation guide was revised and discussed within the multidisciplinary team including doctors, nurses, sociologists, psychologists, health scientists, and nursing scientists. The revision aimed at identifying key topics in the field of end-of-life care with relevance to patients diagnosed with advanced non-oncological chronic-disease.

#### Workshops

**Fig. 1:**
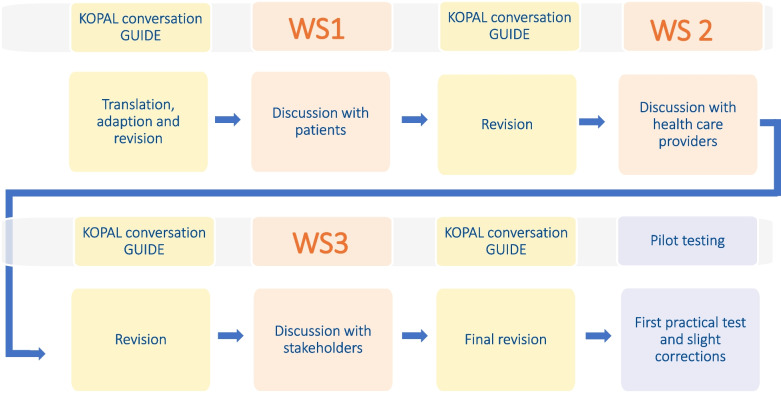
Work flow

The KOPAL conversation guide was subsequently discussed in three expert workshops (WS) with patients diagnosed with COPD or CHF (WS 1), health care providers in the field of primary and palliative care, cardiology, and pneumology (WS 2), and stakeholders (i.e. researchers, health care providers, patients’ representatives, WS 3). Since the guide was built on the well accepted PEPSI COLA aide memoire, special focus was put on potentially unmet needs for non-oncological long-term illness with slow progress. The KOPAL conversation guide was revised after every WS. The revised version provided the basis for discussion of the next WS (see Fig. 1).

#### Participants

WS 1. Participants were recruited in an outpatient clinic for primary care via information material or by direct invitation of physicians. Eligible participants had a documented diagnosis of an advanced COPD or CHF, or were relatives of a person suffering from advanced dementia. WS 2. GPs, cardiologists, and pulmonologists were recruited via previously established institutional and personal professional contacts. Local outpatient PC providers received a written invitation. WS 3. Stakeholders were recruited through different clinics, practices, research institutions, and patients’ organizations in regard to different professions and disciplines. While researchers and participants of WS 1 and 2 met for the first time during the WS, some participants of WS 3 knew each other through previous professional contacts. However, the WS 3 group met in that formation for the first time to discuss the KOPAL conversation guide. Participants of the third WS were also members of the advisory board of the KOPAL study.

The trial is registered on the German clinical trial register (registration number DRKS00017795; first registration 09/01/2020).

#### Discussions

The topics of the KOPAL conversation guide were discussed during three two-hour focus groups (FG) considering the different perspectives (patients/relatives, providers, stakeholders). FG were moderated by two experienced researchers (NP, GM both PhD) and observed by a third researcher (TM, M.A.). At the beginning of each WS participants were introduced to details of the study in general and the KOPAL conversation guide in particular. Each participant received a preliminary version and could refer to details at any time. During the first part three predefined questions led the discussion of each WS: ‘*Which issues do you want to talk about?’, ‘Which issues do you not want to talk about in any way?’, and ‘What is good / bad?—Please explain’*. Participants were asked to imagine themselves in the situation of using the KOPAL conversation guide. In a second step, the KOPAL conversation guide was reviewed with focus on the following questions: *‘Which topic is relevant?’, ‘Which topic is dispensable?’, ‘Which topics / issues should be added?’* All statements were documented immediately, visualised for all participants, and discussed until consent was reached. Discussions were not audiotaped.

After each WS, the KOPAL conversation guide was revised. The revised version served as the basis for discussion in the following WS. Conflicting statements between focus groups were discussed and consented within the research team.

## Results

In total, 4 patients, 7 health care providers, and 5 stakeholders participated in the workshops (WS 1: *n* = 4 patients; WS 2: *n* = 4 healthcare providers; WS 3: *n* = 8 stakeholders). Relatives of patients with dementia could not be recruited. Their perspectives were taken into account by participation of a respective patient representative in the third workshop. All workshops were conducted at the department of the consortium leader.

### Key topics of the KOPAL conversation guide

The first version of the KOPAL conversation guide covered nine key topics: living with the illness, physical situation, emotional situation, spirituality, sexuality, social situation, information and communication, control and autonomy, emergency management. The topics should cover at least three main issues based on the Ottawa Charta on Health Promotion, the WHO-definition of palliative care, and the S3 guideline of palliative care and are described below in detail [16–18]: (1) Four dimension of the human, (2) patients’ needs, and (3) health related quality of life. (1) The *four dimensions of the human* are central in the holistic approach of palliative care as stated in the S3-guideline Palliative Medicine for cancer patients [18]: *physical dimension* (somatic issues), *psychological dimension* (cognitive and emotional dimension), *social dimension* (relational dimension of human relationships with special focus on relatives), *spiritual dimension* (including experiencing or seeking for individual and covering existential questions, e.g., identity, obligation, hope, love, values (relationship to the family, friends, nature, culture, life itself) and religious aspects (belief, religious practices, relationship to God or transcendent). (2) The S3 guideline for palliative care distinguishes between individual need (a subjective-individual wish or an experienced state of stress of a person combined with the wish for relief) and objective need (an objectively recognisable, comprehensible state of stress of a patient that cannot be remedied by his or her own resources). Resources are defined as material or individual/social capabilities to solve the burdensome situation [18]. (3) Health-related quality of life is a key factor in PC and refers to the patients’ self-assessment regarding physical, psychological, social, and daily aspects of wellbeing and functionality.

The KOPAL conversation guide should be used as an aide memoire in order to cover all potentially relevant issues and should be used in an open manner. However, additionally, some validated standardised scales are provided which are commonly used in palliative care: The German Version of the Distress Thermometer of the National Comprehensive Cancer Network (NCCN Distress Thermometer) [19, 20], the Minimal Documentation System for palliative medicine (MIDOS) [21] and the genogram, provided by the German Association of Palliative Medicine (Deutsche Gesellschaft für Palliativmedizin, DGP) to capture the patients social environment [22] were included with permission of the authors.

### Workshops

After discussion in the WS participants suggested to revise six main aspects of the KOPAL conversation guide. 1) The first version of the guide provided space to document instructions for further care at the end of each key topic. Participants found that this could lead to an increased structure of the conversation between PC nurse and patient. Instead, the conversation should be conducted according to the patient’s preferences and relevancy. PC nurses should be able to document the content of the conversation, relevant agreements and possible recommendations to be presented in the case conference. The corresponding section was renamed to ‘space for further notes’. 2) The patient’s current health care situation should be documented explicitly with special focus on nursing aspects, e.g., does an adequate care level (Pflegegrad) exist, how often does the patient receive nurse home visits, how many physicians are involved? 3) Sexuality was discussed as a relevant topic within PC but should be addressed carefully. Since patients as well as nurses showed reservations, the key topic ‘sexuality’ was renamed to ‘personal situation’ comprising cultural, sexual, and spiritual needs. 4) Participants addressed the patient’s knowledge regarding the course of the disease and their understanding of an emergency situation (including emergency management). Further, patient’s needs for more information should be documented. 5) Regarding the key topic ‘control and autonomy’ patients in decreased health should be asked about where and how they want to be cared for. 6) Finally, patients’ knowledge regarding emergency management should be ascertained, e.g., can patients distinguish between a crisis and an emergency situation and how could they regulate themselves within a crisis (e.g., by using breathing techniques).

Furthermore, participants of the WS suggested to start the conversation by addressing the patient’s current well-being and to end it by asking the patients to state their primary concern based on the conversation. The goal should be to address all key topics but adapt the course of the consultation to the individual situation in order to conduct a personal and patient-orientated talk. Table 1 shows the key topics of the KOPAL conversation guide. For space reasons, however, we have only included the short version in this paper. The full version contains further details in addition to each topic, space for notes, and the scales and the genogram mentioned above. The full version is added as appendix 1 and also freely accessible at https://www.uke.de/kopal.

**Table 1:** Topics of the KOPAL conversation guide

**Introductory question**	*‘How are you feeling today?’*
**Key topics**	**General points to be addressed**
Living with the illness	Current care needs of the patient such as: rehabilitation support, admission to other health facilities, non-medical support (e.g. physiotherapy, social services, nutrition counselling), need for medical aids)
Physical situation	Current physical complaints and needs such as: symptoms, medication (regular or on-demand medication), review of current non-essential treatment, side-effects
Emotional situation	Current emotional complaints and needs such as: restlessness, anxiety, joy, loneliness, coping-strategies
Personal situation	Current cultural, sexual and emotional needs such as: *cultural:* migration background, *sexual:* physical closeness, relationship problems, homosexuality, gender identity, *spiritual:* religion, spiritual needs, pastoral care, meaningful life
Social situation	Current social relations, social activities, social support such as: daily activities, social integration, social activities (e.g. parlour games, walks), social support (e.g. Caritas, Red Cross), coping with daily activities, communication
Information and communication	Current information level und communication needs such as: illness knowledge, course of the illness, emergency needs, shared decision making, practical assistance (e.g. logopaedic, ophthalmology, audiology, translation service, self-help group)
Control and autonomy	Current needs on control and autonomy (advance care planning) such as: living will, power of attorney, treatment plan, care plan near to death, preferred place of care (e.g. care support, hospice service), burial (in Germany with reference to §132g SGB V Gesundheitliche Versorgungsplanung für die letzte Lebensphase)
Emergency management	Arrangements of emergency situations such as: emergency medical form (Ärztlicher Notfallbogen, ÄNo), “do not resuscitate”, emergency service of the Association of Statutory Health Insurance Physicians (KV-Notdienst), emergency home care, list of national and personal emergency numbers / contact numbers
**Final question**	*‘We talked about different issues. What is your main topic or main concern?’*

## Discussion

The final version of the KOPAL conversation guide contains eight key topics: living with the illness, physical situation, emotional situation, personal situation, social situation, information and communication, control and autonomy, emergency management. Each key topic refers to a number of related subtopics listed in each respective thematic section. Providers can use these points as a memory aid and document their assessments as free text. In addition, some specific issues of high relevance are pre-formulated as open or closed questions; answers can be documented using check-box or free text format.

Identifying the onset of a palliative trajectory is difficult, especially in regard to non-oncological diseases such as COPD, CHF and dementia [23]. Valid scales defining the starting point for PC are still missing and various tools to predict the illness trajectory are inaccurate [23–25]. However, a current scoping review concluded that the SPICT™ “appears to be a suitable instrument for initiation of palliative care [26]. The surprise question (*Would I be surprised if this patient died within 12 months?*) was known as a tool of variable accuracy and could be a simple tool to screen patients for PC needs. However, in their recent study comprising six European countries, White et al. [27] refer to a high level of inconsistency amongst GPs and therefore, the Surprise Question seems unsuitable for prognosis. The Double Surprise Question (original Surprise Question *Would I be surprised if this patient died in the next 12 months?* plus *Would I be surprised if this patient is still alive after 12 months?*) was shown to be more accurate to identify PC needs of patients with cancer [28]. However, at a certain stage of the disease the illness trajectory of patients with cancer is easier to predict compared to patients diagnosed with non-oncological diseases. Patients with non-oncological disease might have health care needs on different levels and comprising different topics or dimension over a period of more than one year. It may be arguable whether an assessment should identify the switch from curation to palliation since the provision of primary and (specialist) palliative care should not be a decision between either the one or the other [29]. Maddocks et al. [29] suggest partial integrative palliative care when indicated within the individual illness trajectory e.g., in connection with exacerbation or other decline. The KOPAL conversation guide facilitates to identify the resulting potential needs of patients and/or relatives/informal caregivers and to initiate appropriate measures.

A major problem regarding PC provision is physicians’ reluctance to consider and to discuss PC options. Underlying reasons are reservations on the part of patients or the missing association between non-oncological diseases and dying [24, 25]. Furthermore, there remains a need for improvement regarding the GP team approach, e.g., communication with other health care providers [30].

Considering these difficulties and reservations, the KOPAL conversation guide focusses particularly on potential needs likely to be overseen due to the slow illness progression [31]. These are, amongst others, long-term care grade, home medical equipment, social integration (stigmatisation and isolation over a long period), advance care planning, and emergency management. Hickman et al. described reasons for discordance between advance care planning documentation (Ärztlicher Notfallbogen, ÄNo) and patient preferences [32]. The KOPAL conversation guide provides questions suitable to be raised during the consultation to identify patients’ preferences and foster concordance. Although emergency care planning is well known, e.g., ReSPECT [33], the KOPAL conversation guide focusses not only on resuscitation questions in the emergency situation addressing the emergency physician, but particularly on the communication of options between GP and patient/relative in general, e.g., to avoid calling the emergency service. This requires an advanced discussion of appropriate options, e.g., taking specific medication. In case the conversation reveals any possible previously overlooked physical complaints, not only the suggested scales (MIDOS, Distress Thermometer) but also other validated scales such as IPOS, IPOS Dem (https://pos-pal.org/maix/ipos_in_english.php) or others can support a structured in-depth assessment.

### Strengths and limitations

The KOPAL conversation guide was developed by active involvement of the perspectives of relevant actors (patients, health care providers, and experts). However, only few patients and health care providers agreed to take part in workshops 1 and 2. Conversations were trustful and the homogeneous group composition (patients only and health care providers only) allowed issues to be intensively discussed. In PC, consideration of the perspectives of relatives and informal caregivers is necessary as they play an important role. Unfortunately, the perspectives of relatives and informal caregivers, especially those of patients with dementia, could not be taken into full account as we experienced difficulty in their recruitment. However, this perspective will be part of the qualitative evaluation of the KOPAL study [34].

## Conclusions

With the KOPAL conversation guide we developed a broad evaluation and communication tool for the assessment of PC needs of non-oncological patients. SPHC nurses can apply the guide for PC needs assessment of patients with non-oncological diseases during home visits and to structure and support interprofessional case conference in order to strengthen nursing aspects in general PC. Therefore, it provides a basis for interprofessional care planning and counselling. Furthermore, the KOPAL conversation guide implies the chance to foster timely general or specialist PC including various relevant aspects to meet the goal of PC in general: improving health related quality of life, reducing symptom burden, and enhancing patient and caregiver satisfaction. Applying the KOPAL conversation guide in practice may help to bridge the communicative gap between all general and specialist PC professionals and between professionals and patients.

## Supplementary Information

Supplementary Material 1.

## Data Availability

All data generated or analysed during this study are included in this published article.
