# Supplementary material for: Development of a patient assessment to meet the needs of patients suffering from advanced non-oncological diseases – the KOPAL trial
Source: BMC Prim Care. 2025 Feb 22;26:52. doi: 10.1186/s12875-025-02750-z (PMC11846271; doi:10.1186/s12875-025-02750-z)
Supplement: Supplementary file 1 — Supplementary Material 1. [file 12875_2025_2750_MOESM1_ESM.pdf]

ID Patient⌊ ⌋⌊ ⌋⌊ ⌋⌊ ⌋⌊ ⌋⌊ ⌋⌊ ⌋ Date:⌊ ⌋⌊ ⌋.⌊ ⌋⌊ ⌋.⌊ ⌋⌊ ⌋⌊ ⌋⌊ ⌋

# KOPAL-conversation guide

Dear specialist palliative care nurse,

this conversation guide was designed to help you having a conversation with the patient, that is a perfect preparation for your meeting with the specialized palliative care doctor and the case conference with the specialized palliative care team and the general practitioner of the patient.

The conversation should serve as a possibility to make a comprehensive assessment of the patient and his/her current palliative supply needs. At the individual key topics you can find topic-specific questions, which you should discuss with the patient as well as references to tools that should be used. The answers of the patients and your own impressions can be documented in a freely formulated way or by the checkbox options under the respective questions.

The conversation should have the character of a natural conversation and not like a standardised question-answer-scheme. The conversation guide is <u>not</u> a questionnaire. Nevertheless we ask you to pay attention to the following references.

## Some references for the use of the conversation guide:

1. Please <u>always</u> start the conversation with the first question for the general situation of the patient („How are you feeling today?”).
2. Afterwards please conduct an open conversation with the guiding questions of the first key topic about the actual living with the illness.
3. Subsequent to this section please ask the patient to fill out the “Distress-Thermometer” (Appendix A).
4. The order of the following key topics and questions is not predefined. It is important that you can give a concrete overall assessment for every key topic after the conversation.
5. In a suitable place in the further course of the conversation the questionnaire „MIDOS“ (Appendix B) should be filled out by the patient. The actual position of it is a suggestion and not authoriative.
6. Please <u>always</u> end the conversation with the final question (“We talked about different issues. What is your main topic or main concern?”).

ID Patient └ ┘└ ┘└ ┘└ ┘└ ┘└ ┘└ ┘ Date: └ ┘└ ┘.└ ┘└ ┘.└ ┘└ ┘└ ┘└ ┘

# Overview of the topics that should be addressed during the conversation

## Key topic: Living with the illness

In this section current care needs of the patient are addressed.

This includes: rehabilitation support, admission to other health facilities, non-medical support (e.g. physiotherapy, social services, nutrition counselling, need for medical aids)

## Key topic: Physical situation

In this section current physical complaints and needs are addressed.

This includes: symptoms, medication (regular or on-demand medication), review of current non-essential treatment, side-effects

## Key topic: Emotional situation

In this section current emotional complaints and needs are addressed.

This includes: restlessness, anxiety, joy, loneliness, coping-strategies

## Key topic: Personal situation

In this section current cultural, sexual and emotional needs are addressed. This includes:

cultural: migrations background

sexual: physical closeness, relationship problems, homosexuality, gender identity

spiritual: religion, spiritual needs, pastoral care, meaningful life

## Key topic: Social situation

In this section current social relations, social activities, social support are addressed.

This includes: daily activities, social integration, social activities (e.g. parlour games, walks), social support (e.g. Caritas, Red Cross), coping with daily activities, communication

## Key topic: Information and communication

In this section the current information level und communication needs are addressed.

This includes: illness knowledge, course of the illness, emergency needs, shared decision making, practical assistance (e.g. logopaedic, Ophthalmology, audiology, translation service, self-help group)

## Key topic: Control and autonomy

In this section current needs on control and autonomy (advance care planning) are addressed.

This includes: living will, power of attorney, treatment plan, care plan near to death, preferred place of care (e.g. care support, hospice service), burial (in Germany with reference to §132g SBG Gesundheitliche Versorgungsplanung für die letzte Lebensphase)

## Key topic: Emergency management

In this section arrangements of emergency situations are addressed.

This includes: manual for crisis support (Ärztlicher Notfallbogen, ÄNo), "do not resuscitate", emergency service of the Association of Statutory Health Insurance Physicians (KV-Notdienst), emergency home care, list of national and personal emergency numbers / contact numbers

ID Patient ⌊ ⌋⌊ ⌋⌊ ⌋⌊ ⌋⌊ ⌋⌊ ⌋⌊ ⌋ Date: ⌊ ⌋⌊ ⌋.⌊ ⌋⌊ ⌋.⌊ ⌋⌊ ⌋⌊ ⌋⌊ ⌋

## Space for your notes

 © Institut und Poliklinik für Allgemeinmedizin, Universitätsklinikum Hamburg-Eppendorf, 20245 Hamburg 

ID Patient ⎣ ⎦⎣ ⎦⎣ ⎦⎣ ⎦⎣ ⎦⎣ ⎦⎣ ⎦ Date: ⎣ ⎦⎣ ⎦.⎣ ⎦⎣ ⎦.⎣ ⎦⎣ ⎦⎣ ⎦⎣ ⎦

## Introductory question

| |
|---|
| *At the beginning of the conversation (before any other questions) the current general health situation of the patient should be captured.* |
| „How are you feeling today?“ (Taken from the MIDOS-sheet)<br>☐ very poor ☐ poor ☐ average ☐ good ☐ very good |

## Key topic: Living with the illness

| |
|---|
| In this section current care needs of the patient are addressed.<br>This includes: rehabilitation support, admission to other health facilities, non-medical support (e.g. physiotherapy, social services, nutrition counselling), need for medical aids) |
| 1. How is the patient coping with everyday life? What problems/limitations exist?<br><br>☐ no limitations ☐ small limitations ☐ severe limitations |
| **2. By which therapists, nurses, doctors or other persons is the patient currently supported? Care service, caring relatives?**<br><br>☐ Degree of care available: Degree________ How often does the care service come? ______________<br>☐ further support necessary, by: __________________________________________________<br>☐ sufficient support available |
| 3. What aid does the patient recieve? *(Please also document what you are noticing)*<br><br>☐ further aids necessary, namely: ____________________________________________________<br>☐ sufficent aids available |
| Space for further notes |
| Overall assessment of the living with the illness/ recommendation for action: |
| Survey of the current situation with the help of the “Distress-Thermometer” (Appendix A).<br>*Please give the questionnaire to the patient and ask him/her to fill in completely.* |

## Key topic: Physical situation

In this section current physical complaints and needs are addressed.

<u>This includes:</u> symptoms, medication (regular or on-demand medication), review of current non-essential treatment, side-effects

**4. Which primary physical complaints does the patient have?**
*(ask openly first )*

☐ no complaints ☐ small complaints ☐ severe complaints

**5. In what way is the patient limited because of his/her physical complaints?**

☐ no complaints ☐ small complaints ☐ severe complaints

**6. What did the patient do in the past to deal with the complaints?**

**7. How does the patient cope with taking medication?**

| | | |
|---|---|---|
| Does the medication/treatment help? | ☐ yes | ☐ no |
| Is there a medication shedule? | ☐ yes | ☐ no |
| ↳ If so, is it up to date an does the patient understand it? | ☐ yes | ☐ no |
| Does the patient take medications, which not have been prescribed? | ☐ yes | ☐ no |

If so, which (not prescribed) medications does the patient take?

**Space for further notes**

Overall assessment of the physical situation/ recommendation for action:

ID Patient └ ┘└ ┘└ ┘└ ┘└ ┘└ ┘└ ┘     Date: └ ┘└ ┘.└ ┘└ ┘.└ ┘└ ┘└ ┘└ ┘

## 🖋 Survey of the physical complaints with the help of the MIDOS-Fragebogen (Appendix B)

*Please give the questionnaire "MIDOS" to the patient and ask him/her to fill in completely.*

## Key topic: Emotional situation

| |
|---|
| In this section current emotional complaints and needs are addressed.<br>This includes: restlessness, anxiety, joy, loneliness, coping-strategies |
| 8. What concerns the patient the most at the moment? |
| 9. What brings joy to the patient? |
| 10. How does the patient cope with stressful situations?<br><br>☐ sufficient coping-strategies available     ☐ no sufficient coping-strategies available |
| Space for further notes |
| Overall assessment of the emotional situation/ recommendation for action: |

ID Patient ⎣ ⎦⎣ ⎦⎣ ⎦⎣ ⎦⎣ ⎦⎣ ⎦⎣ ⎦ Date: ⎣ ⎦⎣ ⎦.⎣ ⎦⎣ ⎦.⎣ ⎦⎣ ⎦⎣ ⎦⎣ ⎦

## Key topic: Personal situation

| |
|---|
| In this section current cultural, sexual and emotional needs are addressed. This includes:<br>**cultural**: migrations background<br>**sexual**: physical closeness, relationship problems, homosexuality, gender identity<br>**spiritual**: religion, spiritual needs, pastoral care, meaningful life |
| 11. Is there something the patient would like to change (cultural, sexual, spiritual)?<br><br>☐ no change requests ☐ change requests |
| 12. (In what way) Does the illness burden the couple relationship? |
| 13. (How) Does the illness have an effect on physical closeness and the sexual situation?<br>Does the patient feel impaired in his/her gender identity? In what way? |
| 14. What hope does the patient have with regard to his/her illness? |

## Continuation personal situation

15. If applicable: Does the patient have the opportunity to extert his/her spirituality/ religion the way he/she wants? ☐ yes ☐ no

16. What would help the patient?

17. With whom is the patient talking about his/her thoughts?

**Space for further notes**

Overall assessment of the personal situation/ recommendation for action:

 © Institut und Poliklinik für Allgemeinmedizin, Universitätsklinikum Hamburg-Eppendorf, 20245 Hamburg 

ID Patient└ ┘└ ┘└ ┘└ ┘└ ┘└ ┘└ ┘ Date:└ ┘└ ┘.└ ┘└ ┘.└ ┘└ ┘└ ┘└ ┘

## Key topic: Social situation

In this section current social relations, social activities, social support are addressed.

This includes: daily activities, social integration, social activities (e.g. parlour games, walks), social support (e.g. Caritas, Red Cross), coping with daily activities, communication

Visualization of the patient's environment on the basis of the Genogram (Appendix C)

*Please discuss the social relations with the patient and illustrate it in the Genogram.*

**18. How ist he patient doing in terms of:**

| | | | |
|---|---|---|---|
| Friends and family? | ☐ good | ☐ good enough | ☐ not good |
| Work and finances? | ☐ good | ☐ good enough | ☐ not good |
| Social activities? | ☐ good | ☐ good enough | ☐ not good |
| Recreation? | ☐ good | ☐ good enough | ☐ not good |

**19. Does the patient get the necessary support by family, friends and acquaintances? Who helps the patient where? Is the communication satisfying for the patient?**

| | | | |
|---|---|---|---|
| Housing? | ☐ yes, by: ______________________ | ☐ no | ☐ not necessary |
| Mobility? | ☐ yes, by: ______________________ | ☐ no | ☐ not necessary |
| Help with medication? | ☐ yes, by: ______________________ | ☐ no | ☐ not necessary |
| Help with reading? | ☐ yes, by: ______________________ | ☐ no | ☐ not necessary |
| Walking? | ☐ yes, by: ______________________ | ☐ no | ☐ not necessary |
| Shopping? | ☐ yes, by: ______________________ | ☐ no | ☐ not necessary |
| Household/cooking? | ☐ yes, by: ______________________ | ☐ no | ☐ not necessary |
| General company? | ☐ yes, by: ______________________ | ☐ no | ☐ not necessary |

**Additions:**

☐ socially well integrated ☐ social support necessary

**Space for further notes**

Overall assessment of the social situation/ recommendation for action:

ID Patient └ ┘└ ┘└ ┘└ ┘└ ┘└ ┘└ ┘ Date: └ ┘└ ┘.└ ┘└ ┘.└ ┘└ ┘└ ┘└ ┘

## Key topic: Information and communication

| |
|---|
| In this section the current information level und communication needs are addressed.<br>This includes: illness knowledge, course of the illness, emergency needs, shared decision making, practical assistance (e.g. logopaedic, Ophthalmology, audiology, translation service, self-help group) |
| **20. Is there something that is unclear for the patient with regard to his/her illness? If yes, what is it?**<br><br>☐ feels adequately informed ☐ whishes more information ☐ needs more information |
| **21. Did the patient have an impact on the choice/possibility of the treatment?**<br><br>☐ yes ☐ partly ☐ no |
| **22. Would it be helpful for the patient to talk about his/her illness to other people with the same illness?**<br><br>☐ yes, exchange desired ☐ unsure ☐ no, no exchange desired |
| **Space for further notes** |
| Overall assesment of the informedness and communicative situation/ recommendation for action: |

## Key topic: Control and autonomy

In this section current needs on control and autonomy (advance care planning) are addressed.
This includes: living will, power of attorney, treatment plan, care plan near to death, preferred place of care (e.g. care support, hospice service), burial (in Germany with reference to §132g SBG Gesundheitliche Versorgungsplanung für die letzte Lebensphase)

**23. What future treatment whishes are there, especially for the case that the patient could not be asked (e.g. in case of unconsciousness or fainting)?**

**24. Does the patient want to talk about how he/she would like to be treated if an acute emergency situation should arise and he/she is not responsive at this moment?**

☐ no ☐ yes ☐ conversation with GP necessary

**25. How/where the patient wants to be cared for in case his/her health situation deteriorates?** (This could be a sensitive topic for family caregivers)

Need for advice on care topis? ☐ yes ☐ no

**26. Has the patient thought about what should happen (with him/her) after his/her death?**

Need for conversation about the topic of living wills or health care proxies? ☐ yes ☐ no

**Space for further notes**

Overall assessment of the situation of control and autonomy / recommendation for action:

 © Institut und Poliklinik für Allgemeinmedizin, Universitätsklinikum Hamburg-Eppendorf, 20245 Hamburg 

## Key topic: Emergency management

In this section arrangements of emergency situations are addressed.

This includes: manual for crisis support (Ärztlicher Notfallbogen; ÄNo), "do not resuscitate", emergency service of the Association of Statutory Health Insurance Physicians (KV-Notdienst), emergency home care, list of national and personal emergency numbers / contact numbers

27. Does the patient (and his/her relatives) know when there is an emergency or when a situation is an emergency situation?

28. Does the patient (and his/her relatives) know who can be contacted in case of more severe symptoms and insecurity? Is there emergency medication or recommendations for action in an emergency situation? Which agreements are there?

☐ no emergency management available ☐ emergency management necessary ☐ emergency management available

**Space for further notes:**

Overall assessment of the emergency management/ recommendation for action:

## Final question:

*At the end of the conversation (following all other questions) the central topic from the patient's perspective should be inquired.*

"We talked about different issues. What is your main topic or main concern?"

Principles and materials for The Gold Standards Framework © K Thomas, the National GSF Centre 2003 - 2019. Used with permission from the National GSF Centre in End of Life Care Please also add our website address where the copyright licensing policy is available; http://www.goldstandardsframework.org.uk/
Deutsche Version adaptiert im Rahmen der KOPAL-Studie © Institut und Poliklinik für Allgemeinmedizin, Universitätsklinikum Hamburg-Eppendorf; www.uke.de/ipa

ID Patient ⌊ ⌋⌊ ⌋⌊ ⌋⌊ ⌋⌊ ⌋⌊ ⌋⌊ ⌋ Date: ⌊ ⌋⌊ ⌋.⌊ ⌋⌊ ⌋.⌊ ⌋⌊ ⌋⌊ ⌋⌊ ⌋

## Final assessment of the conversation

| |
|---|
| 1. Note why some topics have been left blank. |
| ☐ Not enough time ☐ Conversation too exhausting ☐ Topics are shamefaced |
| 2. Note if you deviate from the conversation guide and, if so, where and why. |
| |
| 3. Note if you carried out interventions and, if so, which. |
| |
| 4. Overall assessment from the perspective of a specialized palliative care nurse. |
| ☐ There is a need for specialized palliative care. ☐ There is no need for specialized palliative care. |
| The preliminary interview was conducted ☐ in person at the place of residence ☐ by telephone |


© Institut und Poliklinik für Allgemeinmedizin, Universitätsklinikum Hamburg-Eppendorf, 20245 Hamburg

ID Patient |_|_|_|_|_|_|_| Date: |_|_|.|_|_|.|_|_|_|_|

## Anhang A - Distress Thermometer

**Anleitung:**

**ERSTENS:** Bitte kreisen Sie die Zahl ein (0-10), die am besten beschreibt, wie belastet Sie sich in der letzten Woche einschließlich heute gefühlt haben.

**Extrem belastet**

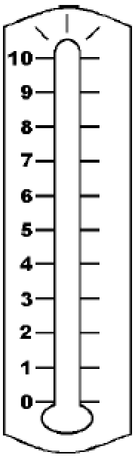


**Gar nicht belastet**

**ZWEITENS:** Bitte geben Sie an, ob Sie in einem der nachfolgenden Bereiche in der letzten Woche einschließlich heute Probleme hatten. Kreuzen Sie für jeden Bereich JA oder NEIN an.

| JA | NEIN | | JA | NEIN | |
|---|---|---|---|---|---|
| | | **Praktische Probleme** | | | **Körperliche Probleme** |
| ❍ | ❍ | Wohnsituation | ❍ | ❍ | Schmerzen |
| ❍ | ❍ | Versicherung | ❍ | ❍ | Übelkeit |
| ❍ | ❍ | Arbeit/Schule | ❍ | ❍ | Erschöpfung |
| ❍ | ❍ | Beförderung (Transport) | ❍ | ❍ | Schlaf |
| ❍ | ❍ | Kinderbetreuung | ❍ | ❍ | Bewegung/Mobilität |
| | | | ❍ | ❍ | Waschen, Ankleiden |
| | | **Familiäre Probleme** | ❍ | ❍ | Äußeres Erscheinungsbild |
| ❍ | ❍ | Im Umgang mit dem Partner | ❍ | ❍ | Atmung |
| ❍ | ❍ | Im Umgang mit den Kindern | ❍ | ❍ | Entzündungen im Mundbereich |
| | | | ❍ | ❍ | Essen/Ernährung |
| | | **Emotionale Probleme** | ❍ | ❍ | Verdauungsstörungen |
| ❍ | ❍ | Sorgen | ❍ | ❍ | Verstopfung |
| ❍ | ❍ | Ängste | ❍ | ❍ | Durchfall |
| ❍ | ❍ | Traurigkeit | ❍ | ❍ | Veränderungen beim Wasser lassen |
| ❍ | ❍ | Depression | ❍ | ❍ | Fieber |
| ❍ | ❍ | Nervosität | ❍ | ❍ | Trockene/juckende Haut |
| | | | ❍ | ❍ | Trockene/verstopfte Nase |
| | | **Spirituelle/religiöse Belange** | ❍ | ❍ | Kribbeln in Händen/Füßen |
| ❍ | ❍ | In Bezug auf Gott | ❍ | ❍ | Angeschwollen/aufgedunsen fühlen |
| ❍ | ❍ | Verlust des Glaubens | ❍ | ❍ | Sexuelle Probleme |

**Sonstige Probleme**: ______________________________

NCCN 1.2006 Distress Management Guideline, © National Comprehensive Cancer Network.
Deutsche Version: Mehnert et al. 2006, Institut und Poliklinik für Medizinische Psychologie, Universitätsklinikum Hamburg-Eppendorf

ID Patient⎿ ⏌⎿ ⏌⎿ ⏌⎿ ⏌⎿ ⏌⎿ ⏌⎿ ⏌    Date:⎿ ⏌⎿ ⏌.⎿ ⏌⎿ ⏌.⎿ ⏌⎿ ⏌⎿ ⏌⎿ ⏌

Anhang B - MIDOS

## Minimales Dokumentationssystem MIDOS zu belastenden Symptomen

| **M1.** Bitte kreuzen Sie an, wie **stark** heute **Ihre Beschwerden** sind. | | | | |
|---|---|---|---|---|
| **Schmerz** | ☐ keine | ☐ leichte | ☐ mittlere | ☐ starke Schmerzen |
| **Übelkeit** | ☐ keine | ☐ leichte | ☐ mittlere | ☐ starke Übelkeit |
| **Erbrechen** | ☐ kein | ☐ leichtes | ☐ mittleres | ☐ starkes Erbrechen |
| **Luftnot** | ☐ keine | ☐ leichte | ☐ mittlere | ☐ starke Luftnot |
| **Verstopfung** | ☐ keine | ☐ leichte | ☐ mittlere | ☐ starke Verstopfung |
| **Schwäche** | ☐ keine | ☐ leichte | ☐ mittlere | ☐ starke Schwäche |
| **Appetitmangel** | ☐ kein | ☐ leichter | ☐ mittlerer | ☐ starker Appetitmangel |
| **Müdigkeit** | ☐ keine | ☐ leichte | ☐ mittlere | ☐ starke Müdigkeit |
| **Depressivität** | ☐ keine | ☐ leichte | ☐ mittlere | ☐ starke Depressivität |
| **Angst** | ☐ keine | ☐ leichte | ☐ mittlere | ☐ starke Angst |
| **Andere:** | ☐ keine | ☐ leichte | ☐ mittlere | ☐ starke |
| **Andere:** | ☐ keine | ☐ leichte | ☐ mittlere | ☐ starke |

**M2.** Bitte kreuzen Sie an, wie Sie sich heute **fühlen**:

☐ sehr schlecht ☐ schlecht ☐ mittel ☐ gut ☐ sehr gut

**Befinden**

**M4.** Selbsterfassung **nicht möglich** wegen:

☐ Sprachproblemen ☐ Schwäche ☐ Kognitiven Störungen ☐ Patient lehnt ab

© Klinik für Palliativmedizin, Universitätsklinikum Bonn, D-53127 Bonn

ID Patient ⌊ ⌋⌊ ⌋⌊ ⌋⌊ ⌋⌊ ⌋⌊ ⌋⌊ ⌋ Date: ⌊ ⌋⌊ ⌋.⌊ ⌋⌊ ⌋.⌊ ⌋⌊ ⌋⌊ ⌋⌊ ⌋

Anhang C

*„Die Karte ist nicht die Landschaft"*

# Patientenumfeld

Name | Datum |

| | |
|---|---|
| Professionelle Helfer | Familie |
| Vereine/Kirche/Arbeit... | Freunde/Bekannte |

## Legende Genogramm

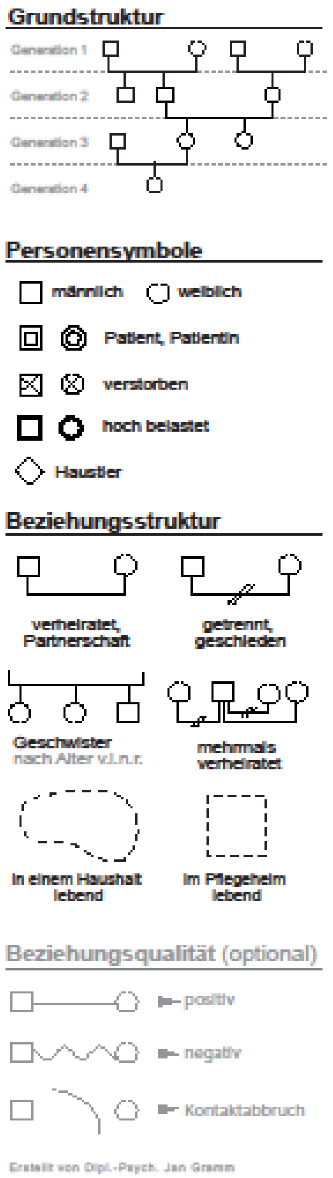


© Deutsche Gesellschaft für Palliativmedizin, Autor: Jan Gramm
